# Long latency between GAD-antibody detection and development of limbic encephalitis – a case report

**DOI:** 10.1186/s12883-015-0435-9

**Published:** 2015-09-30

**Authors:** Susanne Fauser, Ingo Uttner, Helena Ariño, Werner A. Scherbaum, Albert Saiz, Jan Lewerenz

**Affiliations:** Department of Neurology, University Ulm, Oberer Eselsberg 45, 89081 Ulm, Germany; Epilepsiezentrum Bethel, Maraweg, 2133617 Bielefeld, Germany; Service of Neurology, Institut d’Investigació Biomèdica August Pi i Sunyer (IDIBAPS), Hospital Clínic, Villarroel 170, Barcelona, 08036 Spain; Heinrich-Heine University, University Hospital, Moorenstrasse 5, 40225 Duesseldorf, Germany

**Keywords:** Clinical manifestation, GAD antibodies, Limbic encephalitis, Pathogenesis

## Abstract

**Background:**

In the pathogenesis of limbic encephalitis other promoting factors besides the pure existence of autoantibodies are increasingly discussed to play a significant role. This is to our knowledge the first described patient in whom the presence of autoantibodies precedes the manifestation of limbic encephalitis for many years.

**Case presentation:**

At the age of 38 years, in the serum of a patient with polyendocrine autoimmunity high titers of cytoplasmic islet cell antibodies and of anti-glutamate decarboxylase (GAD) 65 antibodies were observed as an incidential finding, GAD67 antibodies were negative at that time. After a latency of 18 years, she manifested with refractory temporal lobe epilepsy most likely due to autoimmune limbic encephalitis. After epilepsy onset, the patient underwent magnetic resonance imaging (MRI), electroencephalography, cerebrospinal fluid (CSF), serum and neuropsychological investigations during a follow-up period of 8 years. A pharmacoresistent epilepsy with seizure onset from the right temporal lobe and declarative memory deficits were observed affecting primarily the recall of verbal informations. MRI showed a slightly increased signal in the right amygdala without progression. GAD antibodies could be detected in serum (titre 1: 1000) and CSF (titre 1:1) by immunofluorescence. Both, GAD65 and GAD67 antibodies were observed in cell-based assays.

**Conclusions:**

It can be assumed that in addition to a pre-existing systemic T-cell response associated with the longstanding polyendocrine autoimmunity, a delayed intrathecal autoimmunity developed leading to limbic encephalitis. This change might be reflected by the development of GAD67 antibodies in our patient. Besides the contribution of this case report to a better understandig of the pathomechanisms for the development of central nervous system (CNS) autoimmunity, it also has a clinical impact as early treatment of GAD antibody-associated CNS disorders has a better prognosis. Therefore, vigilance for symptoms indicating GAD antibody-associated CNS autoimmunity is mandatory in patients with GAD antibody-associated endocrine dysfunction.

## Background

Limbic encephalitis describes a heterogenous spectrum of neurological disorders characterized by mostly subacute onset and progressive mnestic deficits, epileptic seizures and psychiatric disturbances such as depression and psychosis. A variety of autoantibodies against brain antigens has been detected in association with limbic encephalitis. These include onconeuronal antibodies against intracellular proteins (Hu, Ma2, CV2) in the context with malignant tumors [1] and a multitude of other distinct pathogenic autoantibodies against surface protein receptors in the absence or presence of tumors [2] .

Anti-glutamic acid decarboxylase (GAD) antibodies occupy an intermediate position, as they are directed against an intracellular antigen but are not associated with malignant tumors in the majority of cases [2]. Two distinct GAD isoforms exist in humans, GAD65 and GAD67, which are encoded by different genes and share approximately 75 % amino acid sequence identity [3]. Both isoforms are expressed in islet cells and GABAergic neurons, although GAD65 at higher levels. The molecular nature of GAD antibodies is partly distinct in different clinical conditions [4]. Patients with GAD antibodies manifest with different clinical disorders, either with or without neurological dysfunction. Non-neurological disorders comprise type 1 diabetes mellitus alone or polyendocrine autoimmunity with or without diabetes [5, 6] while neurological disorders associated with GAD antibodies include stiff person syndrome (SPS), cerebellar ataxia and limbic encephalitis sometimes presenting as therapy refractory temporal lobe epilepsy [7, 8]. While GAD antibodies in autoimmune endocrinopathies recognize conformational epitopes on GAD65 and the infrequent recognition of GAD67 isoform is assumed as cross-reactivity phenomenon [9], the antibodies in SPS and cerebellar ataxia recognize linear epitopes on GAD65 so they can be detected by Western blotting and frequently recognize GAD67 [10, 11, 12, 13]. Whereas a direct pathogenic mechanism of autoantibodies was demonstrated for antibodies to the N-methyl-D-aspartate receptor (NMDAR antibodies) [14] and can be assumed for limbic encephalitis associated with others surface protein autoantibodies [2], in cases with antibodies against intracellular antigens including GAD antibodies, the toxic effect on the central nervous system (CNS) is more probably mediated by cytotoxic T cells [2]. However, even in autoimmune encephalitis associated with NMDAR antibodies, it has been recently shown that an intrathecal antibody synthesis can persist for a long time after remission of acute NMDAR encephalitis [15]. Thus, other promoting factors seem to be essential to establish the clinical phenotype. Blood brain barrier function and antibody affinity have been discussed as such further important promoting factors.

Here, we report the first patient with limbic encephalitis associated with antibodies to GAD in whom these antibodies had already been detected 18 years *before* the patient became symptomatic with epileptic seizures and mnestic deficits. This case report adds to the medical literature that in longstanding polyendocrine autoimmunity a delayed intrathecal spread is possible leading many years later to limbic encephalitis.

## Case presentation

At the age of 38 years, the female patient participated as a healthy control in scientific studies concerning cytoplasmic islet cell antibodies (ICA) in insulin-dependent diabetes using an indirect immunofluorescence tests on cryostat sections of human pancreas [16–18]. At that time she neither suffered from diabetes mellitus nor presented with any neurological symptoms. However, at the same time, she was diagnosed for autoimmune thyroiditis with high-titre antibodies to thyroid microsomal antigens (although hypothyreoidism had already been known for several years and treated with L-thyroxin), she had vitiligo and antibodies to gastric parietal cells as well as antibodies to the intrinsic factor. At that time - as an incidential finding - high titers of ICA (reportedly >1:128, immunefluorescence on human pancreas) and GAD antibodies were observed in the patient’s serum. The GAD antibodies were directed to the GAD65 antigen only. Antibodies to GAD67 were negative at that time [5]. The ICA reactivity could not be eliminated by preincubation with GAD65 indicating additional antigens against which the ICA was directed [17].

At the age of 56 years, the patient presented with first epileptic seizures. Seizure semiology consisted mainly in simple partial seizures with tightness in the chest accompanied by anxiety resembling angina pectoris. These seizures lasted for 0.5 to 3 min. During most of these seizures, she was adequately responsive to verbal and non-verbal commands. Sometimes, there was a transition to complex partial seizures of temporal semiology. Reportedly, she was unresponsive and appeared helpless. Moreover, she showed fumbling manual automatisms for about 2 to 3 min. The patient had amnesia for these episodes. Additionally she reported acoustic hallucinations in terms of a perception of music/melodies. Generalised tonic-clonic seizures never occurred. Seizure frequency was several simple partial and complex partial seizures per month. Simultaneously with epilepsy onset, the patient complained of considerable memory impairment. A first neuropsychological examination revealed significant episodic memory deficits that affected primarily the recall of verbal information. The figural episodic memory as well as primary verbal memory and verbal fluency were only slightly reduced. Information processing speed, divided and selective attention were in the normal range. Initial cerebrospinal fluid (CSF) examination at the age of 56 was normal (0 leucocyte, total protein 324 mg/l, lactate 1,6 mmol/l, oligoclonal and oligoclonal IgG n serum and CSF negative).

In the cerebral magnetic resonance imaging (MRI) performed three years later, a slight T2-hyperintensity of the right amygdala without increased volume was observed.

**Fig. 1:**
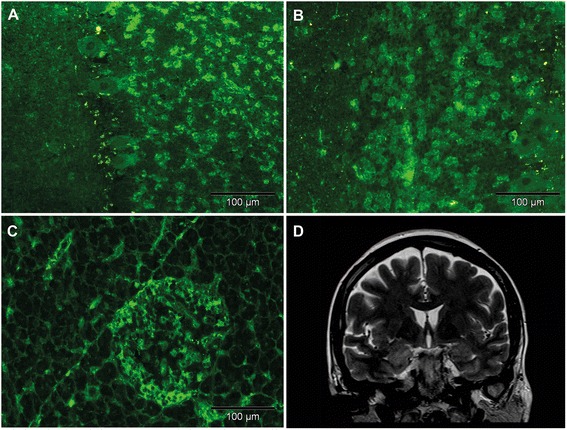
Immunofluorescence findings and magnetic resonance imaging in our case of GAD antibody-associated limbic encephalitis. **a**-**c** Micrographs showing immunofluorescence using the patient’s serum diluted 1:100 (**a**/**c**) or CSF diluted 1:1 (**b**) on primate cerebellum (**a**/**b**) or pancreas (**c**) (bar = 100 μm). Note the typical fluorescence pattern for GAD antibodies with prominent fluorescence in the granule cell layer and a subset of islet cells and less prominent signal in Purkinje cells. **d** T2-weighted magnetic resonance imaging demonstrates a slight signal hyperintensity of the right amygdala/anterior hippocampus compatible with the diagnosis of limbic encephalitis

At the age of 60, video-electroencephalography (EEG) monitoring was performed. Interictal epileptiform discharges were seen in the right temporo-anterior region. One habitual complex partial seizure could be registered with EEG onset also over the right temporal lobe. In a neuropsychological follow-up performed, a substantial worsening of the figural episodic memory performance was found, whereas episodic verbal memory and verbal fluency presented nearly normal. Repeated CSF analysis at the age of 60 again revealed a normal cell count (1 cells/μl), CSF/blood barrier function (total protein 271 mg/l, CSF/serum albumin ratio 3.0 x 10^−3^) and absent intrathecal immunoglobulin synthesis. However, GAD antibodies were highly positive in both serum and CSF when tested by a GAD65 ELISA (serum 15.800 U/ml, CSF 48 U/ml) as described [7] or using immunofluorescence on primate cerebellum (serum 1:1000, CSF 1:1) (Fig. 1a-c) and rat brain (serum 1:64,000, CSF 1:8) [7]. GAD antibodies in serum recognized a linear GAD65 epitope, as suggested by the positive reactivity in immunoblots using recombinant GAD65 protein [13]. Concomitant antibodies against GAD67 (serum 1:40, CSF 1:5) were found in serum and CSF using a cell-based assay [19]. Indirect immunofluorescence using both rat brain and primate cerebellum, pancreas and gut did not reveal the presence of antibodies at relevant titres to neuronal antigens other than GAD. Specific subtests could exclude NMDAR, CASPR2, LGI1, AMPAR, GABA_B_R and GABA_A_R antibodies in serum and CSF and GlyR, Aquaporin 4, Hu, Yo, Ri, CV2, Tr, Ma1/2, Zic4 antibodies in serum.

In the following years, the epilepsy took a pharmacoresistent course. The patient was treated by levetiracetam, lamotrigine and zonisamide without relevant improvement. The MRI abnormality remained unchanged over an observation time of 3 years (Fig. 1d). A further neuropsychological assessment at the age of 62 revealed again mnestic deficits mainly concerning the recall of verbal episodic information and – to a less severe degree - the primary verbal memory while non-verbal memory functions had improved. Moreover, the patient presented with deficits in the selective attention and with emotional lability as well as moderate depressive symptomes. However, during the course of the disease the patient remained able to work. At the age of 63, the patient received two cycle of high-dose methylprednisolone (1 g /daily for 5 consecutive days every four months). In parallel, the seizure frequency decreased considerably to less than once per month although in parallel her antiepileptic therapy was complemented by perampanel.

Until present, the patient has not developed diabetes mellitus.

Based on the clinical findings of late-onset temporal lobe epilepsy with a pharmacoresistant course, impairment of both, verbal and nonverbal declarative memory, a slight increase of signal intensity in the right amygdala without hippocampal atrophy or sclerosis and high GAD65 and positive GAD67 antibodies in serum and CSF, a GAD antibody-associated limbic encephalitis was diagnosed.

## Conclusion

Initially, GAD antibodies in our patient were only associated with endocrine autoimmunity for many years. Thus, our case illustrates that besides the pure presence of the autoantibodies other promoting factors must play a role in the pathogenesis of limbic encephalitis. Whereas complete recovery from symptoms of autoimmune encephalitis has been described despite the persistence of autoantibodies [15], our patient is to our knowledge the first in whom the presence of autoantibodies preceded the manifestation of limbic encephalitis for many years. Although autoimmune encephalitis in terms of GAD autoimmunity cannot be proven in our patient, there are many arguments in favor of this hypothesis. First, high antibody levels were found in the range previously described associated with GAD antibody-associated neurological syndromes [7], which detected a linear epitope as found in other GAD antibody-associated CNS disorders [13]. Second, GAD67 antibodies were positive in the CSF, a finding invariably present in patients with GAD antibody-associated disorders of the CNS [19]. Third, the clinical course with prominent pharmacoresistant epilepsy and less apparent psychiatric and cognitive disturbances is typical of GAD antibody-positive limbic encephalitis [8]. Fourth, in cranial MRI a slight increase of signal intensity was seen in the right amygdala without enlarged volume or hippocampal sclerosis, which remained without progression over three years. Thus, a low-grade brain tumor, mesial temporal sclerosis or a dysplasia is unlikely to exist in this case.

How could the long latency between the appearance of GAD antibodies and the clinical manifestation of limbic encephalitis be explained? In NMDAR encephalitis, in contrast to GAD antibody-associated syndromes, the NMDAR antibodies play a pathophysiologically relevant role [14]. Here, a spread from systemic to intrathecal autoimmunity against NMDARs is hypothesized to trigger the onset of the encephalitis [20]. In patients with antibodies against intracellular antigens, including those with GAD antibodies, a higher CD8/CD3 ratio and more frequent appositions of granzyme-B(+) cytotoxic T cells to neurons were found compared to the patients with surface antigens [21]. In analogy with NMDAR encephalitis, however, it can be assumed that in addition to the pre-existing systemic T cell response associated with the longstanding polyendocrine autoimmunity in our patient, a delayed intrathecal autoimmunity developed leading to limbic encephalitis. This change might be reflected by the development of GAD67 antibodies in our patient. Moreover, a (transient) blood–brain barrier disruption might have played an important part in the reported patient. Alternatively, the epilepsy may not have occurred immediately after tissue damage but after a longer latency period in which remodelling of synapses finally lead to epileptogenesis similar to hippocampal sclerosis.

In conclusion, our case shows that systemic GAD autoimmunity, even after many years, can spread to the CNS. Moreover, our case underlines that the presence of one or more autoimmune disorders is an indicator that epilepsy may be of autoimmune origin [22]. As early treatment of GAD antibody-associated CNS disorders has a better prognosis [19], vigilance for symptoms indicating GAD antibody-associated CNS autoimmunity is mandatory in patients with GAD antibody-associated endocrine dysfunction.

### Consent

Written informed consent was obtained from the patient for publication of this Case report and any accompanying images. A copy of the written consent is available for review by the Editor of this journal.

## Abbreviations

CNS: Central nervous system
CSF: Cerebrospinal fluid
EEG: Electroencephalography
GABA: Gamma-amino-butyric acid
GAD: Anti-glutamate decarboxylase
ICA: Cytoplasmic islet cell antibodies
MRI: Magnetic resonance imaging
NMDA: N-methyl-D-aspartate
NMDAR: N-methyl-D-aspartate receptor
SPS: Stiff person syndrome
